# Epidemiology and timing of seasonal influenza epidemics in the Asia-Pacific region, 2010–2017: implications for influenza vaccination programs

**DOI:** 10.1186/s12889-019-6647-y

**Published:** 2019-03-21

**Authors:** Clotilde El Guerche-Séblain, Saverio Caini, John Paget, Philippe Vanhems, François Schellevis

**Affiliations:** 1 Global Vaccine Epidemiology and Modeling department (VEM), Sanofi Pasteur, Lyon, France; 2 Netherlands Institute for Health Services Research (NIVEL), Utrecht, The Netherlands; 3 Epidemiology and International Health Team, Emergent Pathogens Laboratory, Fondation Mérieux, International Center for Research in Infectiology, National Institute of Health and Medical Research, U1111,National Center of Scientific Research, Mixed Scientific Unit 5308, École Nationale Supérieure de Lyon, Université Claude Bernard Lyon 1, Lyon, France; 4 Department of General Practice & Elderly Care Medicine, Amsterdam Public Health Research Institute, Amsterdam University Medical Centres, Amsterdam, The Netherlands

**Keywords:** Influenza, Epidemiology, Vaccination, Asia-Pacific, FluNet

## Abstract

**Background:**

Description of the epidemiology of influenza is needed to inform influenza vaccination policy. Here we examined influenza virus circulation in countries in the Asia-Pacific region and compared the timing of seasonal epidemics with the timing of influenza vaccination.

**Methods:**

Data were obtained from the World Health Organization (WHO) FluNet database for 2010–2017 for countries in the WHO Asia-Pacific region. Data from countries covering ≥5 consecutive seasons and ≥ 100 influenza positive cases per year were included. Median proportions of cases for each influenza virus type were calculated by country and season. The timing and amplitude of the epidemic peaks were determined by Fourier decomposition. Vaccination timing was considered appropriate for each country if it was recommended ≤4 months before the primary peak of influenza circulation.

**Results:**

Seven hundred eleven thousand seven hundred thirty-four influenza cases were included from 19 countries. Peak circulation coincided with the winter seasons in most countries, although patterns were less clear in some countries in the inter-tropical area due to substantial secondary peaks. Influenza A/H3N2 dominated overall, but proportions of A and B strains varied by year and by country. Influenza B represented 31.4% of all cases. The WHO-recommended timing for influenza vaccination was appropriate in 12 countries. Vaccination timing recommendations were considered inappropriate in Laos, Cambodia, and Thailand, and were inconclusive for India, Sri Lanka, Singapore, and Vietnam due to unclear seasonality of influenza virus circulation.

**Conclusions:**

Influenza virus circulation varied considerably across the Asia-Pacific region with an unusually high burden of influenza B. The recommended timing for vaccination was appropriate in most countries, except for several countries with unclear seasonality, mainly located in the inter-tropical area.

**Electronic supplementary material:**

The online version of this article (10.1186/s12889-019-6647-y) contains supplementary material, which is available to authorized users.

## Background

Seasonal influenza epidemics cause approximately 3 to 5 million cases of severe influenza and about 290,000 to 650,000 respiratory deaths each year globally [1, 2]. Epidemiological data are needed to develop policies and specific measures to control influenza spread. Although extensive epidemiological data exist for temperate regions of Europe and North America, much less is available for the Asia-Pacific region [3], which contains about 60% of the world’s population [4]. The Asia-Pacific region is believed to have a similar burden of influenza to countries with temperate climates, but is considered to be an important source of new viruses and global influenza epidemics due to its large and highly interacting human and animal populations [5]. As in the rest of the world, most influenza illness in the Asia-Pacific region is caused by influenza A viruses. However, influenza A and B co-circulate with varying patterns, and in some seasons influenza B is the dominant strain [3, 5, 6].

In countries with temperate climates, influenza virus circulation generally peaks during the winter months, between late December and February in the Northern Hemisphere and between April and September in the Southern Hemisphere [7]. However, in countries with tropical or subtropical climates, influenza seasonality is more variable with influenza activity observed throughout the year, especially during the rainy seasons [8]. Within the tropical and subtropical zones of the Asia-Pacific region, influenza viruses circulate throughout the year in one of two latitude-dependent circulation patterns. In the first pattern, which occurs in tropical or partially tropical countries (e.g., Bangladesh, Cambodia, Thailand, and Vietnam), influenza virus circulation peaks during the summer monsoon season (usually between July and October); in the second pattern, which tends to occur in countries on or close to the equator (e.g., Indonesia, Malaysia and Singapore), influenza viruses circulate at a stable level throughout the year with no obvious discrete peak [3].

Because several countries in the Asia-Pacific region have more than one type of climate, regions within a country can show different influenza virus activity, making it difficult to optimize the timing of national vaccination programs [3]. Information on the epidemiology and burden of influenza is therefore needed to guide influenza vaccination policies in the Asia-Pacific region, particularly for the few countries without guidelines or recommendations in place [3, 9, 10]. Some studies have identified a need for improved levels of surveillance [3, 6, 11]; however, little is known about the circulation of influenza A and B viruses, the spatial timing of epidemics, and how well influenza vaccination timing anticipates virus circulation. Here, we compared the patterns of influenza circulation in countries in the Asia-Pacific region to assess the timing of seasonal epidemics and the relationship with vaccination timing as recommended by the World Health Organization (WHO).

## Methods

### Data source and extraction

Influenza surveillance data from week 1 of 2010 to week 52 of 2017 were extracted from WHO FluNet [12] for each of the 53 countries included in the WHO Eastern Asia, Southern Asia, South-East Asia, and Oceania-Melanesia-Polynesia influenza transmission zones [13]. WHO FluNet is a web-based data collection and reporting tool of the WHO’s Global Influenza Surveillance and Response System (GISRS) first launched in 1997 [14]. The data are provided remotely by National Influenza Centres of the GISRS and other national influenza reference laboratories collaborating actively with the GISRS, or they are uploaded from WHO regional databases. Data are publicly available and updated weekly.

The following data were extracted: numbers of laboratory-confirmed cases for any influenza virus type or subtype, any A (i.e., regardless of the A subtype), A(H1N1), A(H3N2), A not subtyped, any B, B Victoria lineage, B Yamagata lineage, and B not subtyped. Data from countries were included in the analysis if they were available for at least five consecutive years, with each year having ≥100 laboratory-confirmed influenza cases recorded. For each included country, the number of laboratory-confirmed cases was calculated by week for each type and subtype. The latitude and longitude of each country centroid was obtained from the US Central Intelligence Agency World Fact Book [15]. Maps were generated using mapchart [16].

### Statistical analyses

The timing and amplitude of the primary and secondary influenza epidemic peaks in each country were determined as described previously [17]. Briefly, the weekly number of reported influenza cases was divided by the highest weekly number of cases in each season (January to December), representing the peak activity. The annual, semi-annual, and quarterly harmonics obtained from Fourier decomposition were summed to generate a periodic annual function describing the peak of activity and intensity of the seasonality. The timing of the peaks refers to the month when influenza activity reached its maximum value. The amplitude of the peak(s) was used to quantify the intensity of the seasonality. The primary peak was defined as the highest amplitude during the year. The secondary peak was defined as the second highest amplitude, regardless of whether it occurred before or after the primary peak. Timing of the primary peak of influenza virus circulation resulting from this study and WHO vaccination timing recommendations [18] were compared and categorized as appropriate, inappropriate, or uncertain. Vaccination timing was considered appropriate if it was recommended ≤4 months before the primary peak of influenza virus circulation in 2010–2017. This corresponds to an accepted duration of protection from influenza vaccination [19–23]. Statistical analysis was performed using Stata version 14 (Stata Corp, College Station, TX) and EPIPOI [24].

## Results

### Available data

**Fig. 1:**
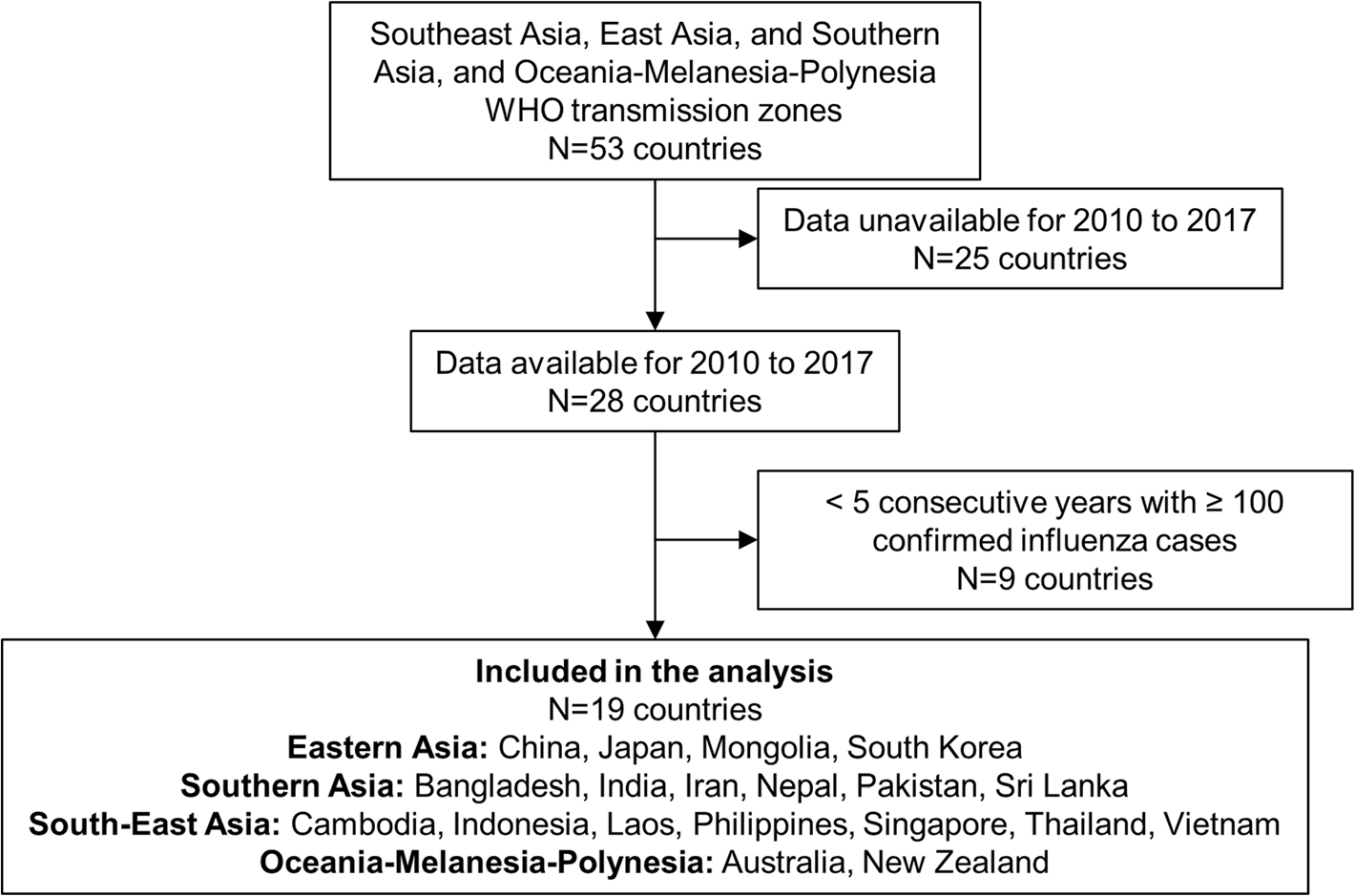
Selection of countries included in the analysis. Influenza surveillance data from week 1 of 2010 to week 52 of 2017 were extracted from FluNet for each of the 53 countries included in the World Health Organization Eastern Asia, Southern Asia, South-East Asia, and Oceania-Melanesia-Polynesia influenza transmission zones. Countries were excluded if they did not have data for at least five consecutive years, with each year having ≥100 laboratory-confirmed influenza cases

**Fig. 2:**
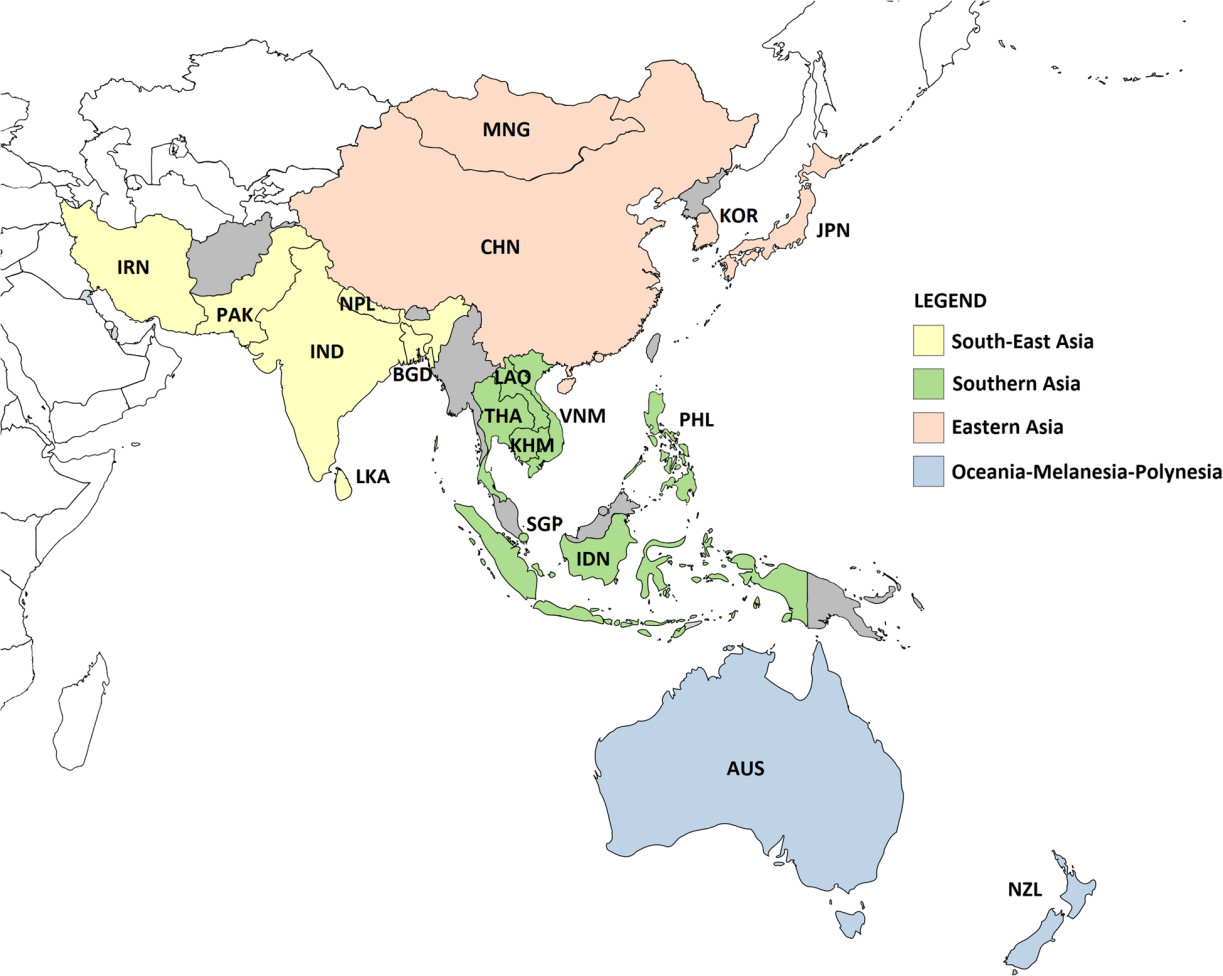
Influenza transmission zones included in the analysis. Countries shaded in grey were not included in the analysis. Abbreviations: AUS = Australia, BGD = Bangladesh, CHN=China, IDN=Indonesia, IND = India, IRN=Iran, JPN = Japan, KHM = Cambodia, KOR = South Korea, LAO = Laos, LKA = Sri Lanka, MNG = Mongolia, NPL = Nepal, NZL = New Zealand, PAK=Pakistan, PHL = Philippines, SGP=Singapore, THA = Thailand, VNM = Vietnam. Map generated using mapchart [16] software

Influenza surveillance data for 2010 to 2017 were available from FluNet for 28 of 53 countries in the WHO Eastern Asia, Southern Asia, South-East Asia, and Oceania-Melanesia-Polynesia influenza transmission zones. Data from nine countries (Afghanistan, Bhutan, Fiji, Malaysia, Maldives, Myanmar, New Caledonia, North Korea, Papua New Guinea) were excluded from the analysis because the data covered less than five consecutive years with ≥100 laboratory-confirmed influenza cases (Fig. 1). Therefore, the study database included influenza surveillance data for 19 countries. The included countries were distributed from latitude 46°N to 41°S and from longitude 53°E to 174°E, and the overall population was 3.98 billion (96.2% of the total population of the Asia-Pacific region) based on 2015 estimates [25] (Fig. 2). Except for Laos (2011–2017), Nepal (2012–2017), and Pakistan (2010–2016), data for each country covered all years in the study period (2010–2017).

### Influenza virus circulation by influenza transmission zone, country and year

Overall, 711,734 laboratory-confirmed influenza cases were reported in the 19 countries included (Table 1). Due to a high number of cases in China (*n* = 473,583; 66.5%), most were located in the Eastern Asia influenza transmission zone. Influenza A dominated overall (68.6%; A/B ratio = 2.18) and in each country during the study period. Influenza B was most common in the South-East Asia region (36.3%; A/B ratio = 1.76) and least common in Southern Asia (23.0%; A/B ratio = 3.35).

**Table 1:** Laboratory-confirmed influenza cases overall and by type for each country between 2010 and 2017

Influenza transmission zone/country	Influenza cases, N (% of total)	n (% of cases)		A/B ratio
		Any influenza A	Any influenza B	
Eastern Asia	563,511 (79.2)	381,783 (67.8)	181,728 (32.2)	2.10
China	473,583 (66.5)	315,130 (66.5)	158,453 (33.5)	1.99
Japan	69,123 (9.7)	53,819 (77.9)	15,304 (22.1)	3.52
Mongolia	4055 (0.6)	2727 (67.3)	1328 (32.7)	2.05
South Korea	16,750 (2.4)	10,107 (60.3)	6643 (39.7)	1.52
Southern Asia	54,400 (7.6)	41,885 (77.0)	12,515 (23.0)	3.35
Bangladesh	5582 (0.8)	3631 (65.0)	1951 (35.0)	1.86
India	21,082 (3.0)	18,024 (85.5)	3058 (14.5)	5.89
Iran	9807 (1.4)	7139 (72.8)	2668 (27.2)	2.68
Nepal ^a^	7863 (1.1)	5590 (71.1)	2273 (28.9)	2.46
Pakistan ^b^	2547 (0.4)	1929 (75.7)	618 (24.3)	3.12
Sri Lanka	7519 (1.1)	5572 (74.1)	1947 (25.9)	2.86
South-East Asia	44,681 (6.3)	28,483 (63.7)	16,198 (36.3)	1.76
Cambodia	4077 (0.6)	2539 (62.3)	1538 (37.7)	1.65
Indonesia	7567 (1.1)	4384 (57.9)	3183 (42.1)	1.38
Laos ^c^	3216 (0.5)	1907 (59.3)	1309 (40.7)	1.46
Philippines	5451 (0.8)	3013 (55.3)	2438 (44.7)	1.24
Singapore	10,317 (1.4)	7441 (72.1)	2876 (27.9)	2.59
Thailand	7860 (1.1)	5190 (66.0)	2670 (34.0)	1.94
Vietnam	6193 (0.9)	4009 (64.7)	2184 (35.3)	1.84
Oceania-Melanesia-Polynesia	49,142 (6.9)	36,112 (73.5)	13,030 (26.5)	2.77
Australia	33,393 (4.7)	25,500 (76.4)	7893 (23.6)	3.23
New-Zealand	15,749 (2.2)	10,612 (67.4)	5137 (32.6)	2.07
Total	711,734 (100.0)	488,263 (68.6)	223,471 (31.4)	2.18

^a^Data were available for 2012–2017

^b^Data were available for 2010–2016

^c^Data were available for 2011–2017

**Fig. 3:**
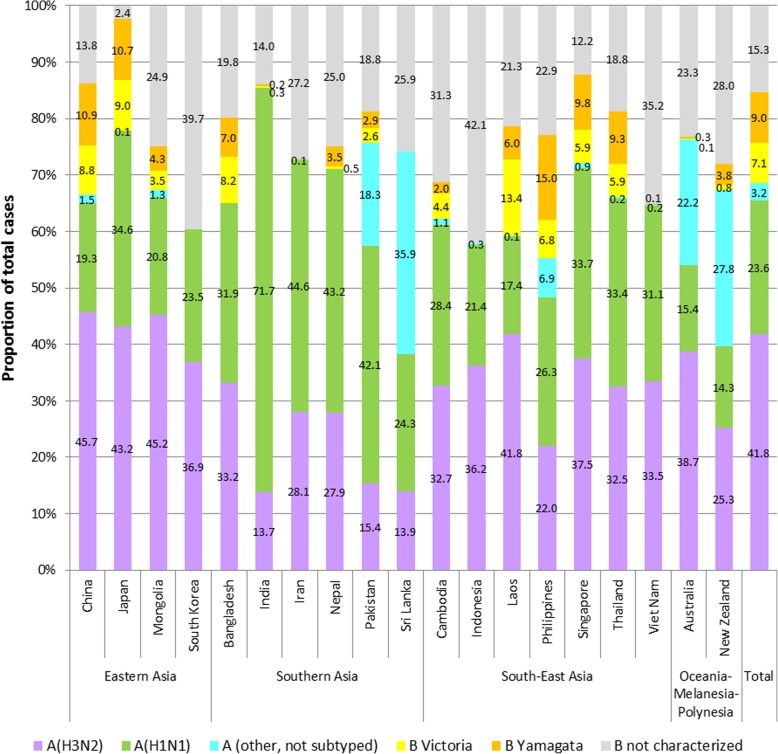
Circulation of influenza strains by country and overall

Strain circulation varied substantially (Fig. 3 and Additional file 1: Table S1). Of the A types, A(H3N2) dominated overall (41.8%), although the dominant A subtype varied among regions and countries. Of the B lineages, Yamagata (9.0%) was more common than Victoria (7.1%) overall, but the lineage that dominated varied by region and country. The proportion of uncharacterized cases (A or B not subtyped) was only 4.6% for influenza A, but 48.8% for influenza B.

**Fig. 4:**
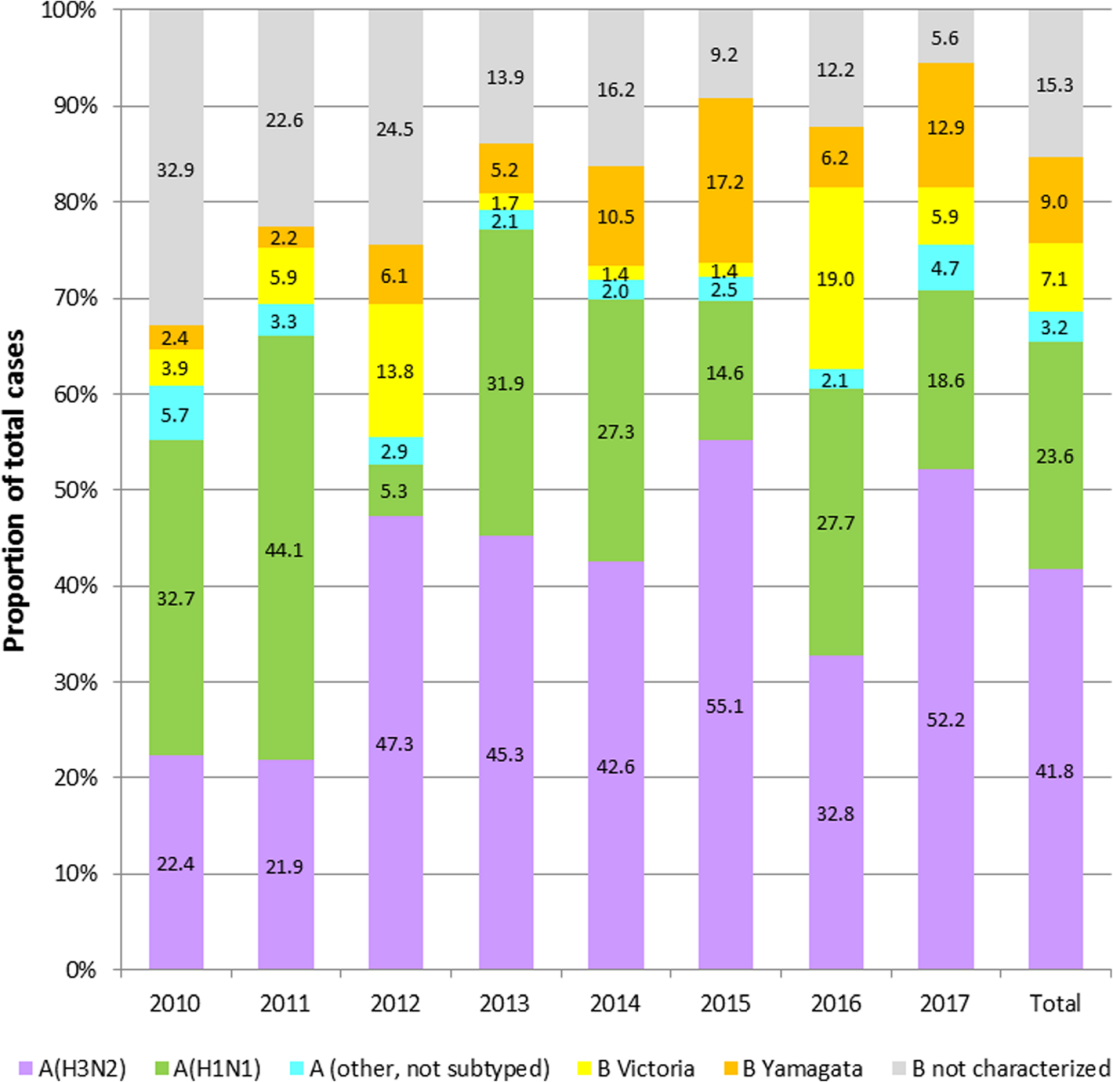
Circulation of influenza strains by year

Influenza A dominated in all years (range, 55.5–79.3%; A/B ratio = 1.25–3.82 (Table 2). In 2010, after the pandemic year, influenza B represented 39.2% of all cases. The proportion of influenza B was highest in 2012 (44.5%; A/B ratio = 1.25) and lowest in 2013 (20.7%; A/B ratio = 3.82). The Yamagata and Victoria lineages of influenza B co-circulated in all years (Fig. 4 and Additional file 2: Table S2). The dominant B lineage was Victoria in 2010, 2011, 2012, and 2016, and Yamagata in 2013, 2014, 2015, and 2017.

**Table 2:** Laboratory-confirmed influenza cases overall and by type for each year between 2010 and 2017

Year	Influenza cases	n (% of cases)		A/B ratio
		Any influenza A	Any influenza B	
2010^a,b^	73,770	44,850 (60.8)	28,920 (39.2)	1.55
2011^a^	48,181	33,401 (69.3)	14,780 (30.7)	2.26
2012	73,707	40,929 (55.5)	32,778 (44.5)	1.25
2013	55,696	44,148 (79.3)	11,548 (20.7)	3.82
2014	99,293	71,346 (71.9)	27,947 (28.1)	2.55
2015	104,312	75,316 (72.2)	28,996 (27.8)	2.60
2016	120,381	75,268 (62.5)	45,113 (37.5)	1.67
2017^c^	136,394	103,005 (75.5)	33,389 (24.5)	3.08
Total	711,734	488,263 (68.6)	22,3471 (31.4)	2.18

^a^Did not include Nepal

^b^Did not include Laos

^c^Did not include Pakistan

### Timing and amplitude of the primary peak of influenza activity

**Fig. 5:**
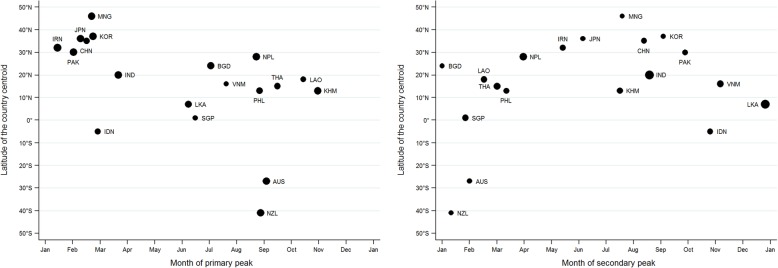
Relationship between the timing and amplitude of the primary peak (left panel) and the secondary peak (right panel) of influenza in each country between 2010 and 2017 and the latitude of the country centroid. The annual, semi-annual, and quarterly harmonics obtained from Fourier decomposition were summed to generate a periodic annual function describing the peak of activity and intensity of the seasonality. Latitude of each country centroid were from the US Central Intelligence Agency World Fact Book [15]. The size of each spot is in proportion to the amplitude for the country. Abbreviations: AUS = Australia, BGD = Bangladesh, CHN=China, IDN=Indonesia, IND = India, IRN=Iran, JPN = Japan, KHM = Cambodia, KOR = South Korea, LAO = Laos, LKA = Sri Lanka, MNG = Mongolia, NPL = Nepal, NZL = New Zealand, PAK=Pakistan, PHL = Philippines, SGP=Singapore, THA = Thailand, VNM = Vietnam

For the northernmost countries (centroid lying latitude ≥30°N: Iran, Pakistan, Japan, China, South Korea, and Mongolia) and countries in the Southern Hemisphere (Australia and New Zealand), the primary peak was in the winter months (i.e., January to March in the Northern Hemisphere and August to September in the Southern Hemisphere) (Table 3 and Fig. 5). For these countries, the amplitude of the primary peak was high (> 100% except in China), while the amplitude of the secondary peak was < 30%.

**Table 3:** Timing and amplitude of influenza peaks by country and comparison with recommended timing of influenza vaccination

Influenza transmission zone/country	Primary peak		Secondary peak		Recommended vaccination timing	Appropriateness of recommended vaccination timing ^a^
	Timing	Amplitude	Timing	Amplitude		
Southern Asia						
Bangladesh	Jul	97.70%	Jan	0.30%	April	Appropriate
India	Mar	105.20%	Aug	87.90%	April	Uncertain
Iran	Jan	111.90%	May	26.90%	October	Appropriate
Nepal	Aug	99.60%	Mar	78.80%	April	Appropriate
Pakistan	Feb	103.10%	Sep	21.10%	October	Appropriate
Sri Lanka	Jun	93.00%	Dec	75.70%	October	Uncertain
Eastern Asia						
China	Feb	85.60%	Aug	26.90%	October	Appropriate
Japan	Feb	103.70%	Jun	12.50%	October	Appropriate
Mongolia	Feb	105.70%	Jul	10.80%	October	Appropriate
South Korea	Feb	107.50%	Sep	15.20%	October	Appropriate
South-East Asia						
Cambodia	Oct	101.20%	Jul	31.50%	April	Not appropriate
Indonesia	Feb	84.00%	Oct	19.50%	October	Appropriate
Laos	Oct	79.40%	Feb	41.90%	April	Not appropriate
Philippines	Aug	93.50%	Mar	24.60%	April	Appropriate
Singapore	Jun	72.60%	Jan	35.90%	October	Uncertain
Thailand	Sep	87.50%	Mar	57.80%	April	Not appropriate
Vietnam	Jul	59.00%	Nov	40.90%	April	Uncertain
Oceania-Melanesia-Polynesia						
Australia	Aug	100.40%	Apr	12.50%	April	Appropriate
New Zealand	Aug	104.30%	Jan	8.70%	April	Appropriate

^a^Vaccination timing was considered appropriate if it was recommended ≤4 months before the primary peak of influenza circulation

For the remaining countries (latitude between 30°N and 10°S), the primary peak occurred throughout the year, and the amplitude was generally lower **(**Fig. 5**).** The lowest amplitude was 59.0% in Vietnam. Most of the secondary peaks for these countries had an amplitude > 30% (except for Bangladesh, Indonesia, and the Philippines) and occurred between January and March or between July and December **(**Fig. 5**).** Nepal and India had distinct influenza virus circulation patterns, in which both the primary and secondary peaks were of substantial amplitude.

### Appropriateness of vaccination timing recommendation

In 12 countries (Bangladesh, Iran, Nepal, Pakistan, China, Japan, Mongolia, South Korea, Indonesia, Philippines, Australia, and New Zealand), the WHO-recommended timing of influenza vaccination was within 4 months before the observed primary peaks of influenza in 2010–2017, and thus considered as appropriate (Table 3). For three countries (Cambodia, Laos, and Thailand), the vaccination timing recommendation was more than 4 months before the primary peaks of influenza in 2010–2017, and so was considered inappropriate. The appropriateness of vaccination timing could not be determined for the remaining four countries (India, Sri Lanka, Singapore, and Vietnam) because these countries had two large peaks of influenza virus circulation separated by several months.

## Discussion

Influenza control depends upon epidemiological data of influenza virus circulation to ensure that vaccination is timed ahead of peak transmission. For most countries, the WHO-recommended timing of vaccination was appropriate for (i.e., within 4 months before) the timing of the influenza peak activity. In contrast, the vaccination timing recommendations in Laos, Cambodia, and Thailand were more than 4 months before the primary peak of influenza activity in these countries, when vaccine-induced immunity is starting to decline [19, 20]. For the remaining four countries (India, Sri Lanka, Singapore, and Vietnam), the appropriateness of vaccination timing could not be determined because of mixed seasonality patterns consisting of two large peaks of influenza separated by several months.

Our results add important information on the timing of influenza epidemics in the Asia-Pacific region, and how well national vaccination programs are timed to precede peak influenza transmission. The seasonality and peaks in circulation we found are generally consistent with those reported for other regions of Southern Asia and South-East Asia [11, 26] and of Iran and Pakistan [17]. However, our analysis builds on these findings by describing influenza epidemics in East Asian countries, including China, Japan, Mongolia, and South Korea, which were not described in these earlier reports. In some cases, our results differed; for example, in India, we found a primary peak in March and a secondary peak in August, whereas Saha et al. reported a single peak in July or August [11] and Hirve et al. reported a primary peak between April and June [26]. Differences between the data in this study and other reports may derive from which surveillance data were used and which years were included. We and Saha et al. [11] used FluNet data exclusively, whereas Hirve et al. used national surveillance data in addition to FluNet data [26]. Moreover, our analysis included data from 2010 to 2017, whereas Saha et al. included 2006 to 2011 and Hirve et al. included 2010 to 2015. These methodological differences, and variability in circulating strains within different influenza seasons, limit comparisons between reports by country.

We also showed that influenza virus circulation patterns varied considerably across the Asia-Pacific region between 2010 and 2017. Overall, influenza B represented 31.4% of cases, which was a higher proportion than reported elsewhere. For example, the overall global median proportion of influenza B was 22.6% for 2000 to 2013 [27], and by region, median proportions were reported to be 21% in Latin America for 2004 to 2012 [28], 17% for Europe for 2000 to 2015 [29], and 23.5% for the Middle East and North Africa for 2000 to 2016 [17]. As reported previously [6, 27], the Victoria and Yamagata lineages of influenza B co-circulated in various proportions between Asia-Pacific sub-regions and between countries in the same sub-region. A higher proportion of influenza B cases were uncharacterized than for influenza A (48.8% vs. 4.6%). This suggests that more resources are needed for B lineage characterization, particularly since influenza B cases represented around one-third of the seasonal influenza burden in most countries. Increasing B-lineage characterization could help inform which B strains to use for trivalent and quadrivalent influenza vaccines in the Asia-Pacific region (and elsewhere considering this region is an important source of new influenza viruses and global epidemics [5]), and would also improve evaluation of vaccine effectiveness by strain subtype in each season.

The WHO recommends that seasonal influenza vaccine should be given prior to the start of the primary period of increased influenza activity [18]. Our study defined appropriate vaccination timing as being a maximum of 4 months before the peak in influenza cases, since several studies have reported that seasonal influenza vaccine protection becomes suboptimal beyond this time [21–23, 30]. However, limited and conflicting data do not allow firm conclusions about the persistence of seroprotection over a defined period. Also, the evolution of influenza virus strains within the same season makes it difficult to distinguish waning vaccine-induced immunity from decreasing match between the vaccine and circulating strains.

Our results suggest – in contrast to recommendations by Hirve et al. [26] – that countries should not be grouped and, instead, national or even regional data within each country should be used to inform when to vaccinate and which vaccines to use. For example, in countries with two peaks of influenza activity or where seasonality is complex or uncertain, offering influenza vaccination within 4 months before both influenza peaks might be most effective. This is consistent with recommendations by Newman et al. [31], who described country-level epidemiology to guide local influenza vaccination programs for the Asia-Pacific region. However, these changes to national influenza vaccination timing could be challenging for some countries in terms of funding, vaccine coverage, and supply – particularly where twice-yearly vaccination is warranted.

This study benefited from the large number of confirmed influenza cases included and the near-complete representation of the Asia-Pacific region’s population. FluNet contains data from surveillance systems in geographically disparate sentinel sites. However, the setting (i.e., community vs. hospital sentinel sites), severity, level of data completeness, and detailed methods for FluNet data collection are not described, and thus limit interpretation of results and comparison between countries. For example, China had the highest proportion of influenza cases (66.5%) consistent with it being the most populous country in the Asia-Pacific region, whereas India, the second-most populous country, had only 3% of all cases.

For countries with a large latitudinal spread, the data may also lack the geographical precision needed to detect sub-national variation in influenza seasonality [32]. Therefore, for countries with mixed influenza seasonality (e.g., India and China), the results should be interpreted with caution. For several countries (i.e., India, Sri Lanka, Singapore, and Vietnam), further in-depth evaluation of spatial timing will be needed to make specific recommendations for the timing of vaccination. This could be addressed through improvements in local surveillance data and their accessibility.

## Conclusions

Our analysis showed that the recommended timing for vaccination was appropriate in most countries, although it was inappropriate or inconclusive for six countries with complex seasonality, most of which are located in the inter-tropical area. Vaccination timing in the Asia-Pacific region may be redefined over time and may be adapted to each country as more local surveillance data become available.

## Additional files

Additional file 1: **Table S1.** Number and proportion of influenza cases in each influenza transmission zone and country overall, by type, and by subtype/lineage for 2010–2017. (PDF 224 kb)

Additional file 2: **Table S2** Number and proportion of influenza cases each year overall, by type, and by subtype/lineage. (PDF 16 kb)

## Abbreviations

GISRS: Global Influenza Surveillance and Response System
WHO: World Health Organization
