# Supplementary material for: Epidemiology and timing of seasonal influenza epidemics in the Asia-Pacific region, 2010–2017: implications for influenza vaccination programs
Source: BMC Public Health. 2019 Mar 21;19:331. doi: 10.1186/s12889-019-6647-y (PMC6429768; doi:10.1186/s12889-019-6647-y)
Supplement: Supplementary file 1 — Table S1. Number and proportion of influenza cases in each influenza transmission zone and country overall, by type, and by subtype/lineage for 2010–2017. (PDF 224 kb) [file 12889_2019_6647_MOESM1_ESM.pdf]

Supplementary Table 1. Number and proportion of influenza cases in each influenza transmission zone and country overall, by type, and by subtype/lineage for 2010–2017

		n (% of influenza cases)							
**Influenza transmission zone/country**	**Any influenza**	**Any A**	**Any B**	**A(H3N2)**	**A(H1N1)**	**A (other, not subtyped)**	**B Victoria**	**B Yamagata**	**B (not characterized)**
Eastern Asia	*563,511*	*381,783 (67.8)*	*181,728 (32.2)*	*254,386 (45.1)*	*120,276 (21.3)*	*7,121 (1.3)*	*47,888 (8.5)*	*59,381 (10.5)*	*74,459 (13.2)*
China	473,583	315,130 (66.5)	158,453 (33.5)	216,505 (45.7)	91,593 (19.3)	7,032 (1.5)	41,527 (8.8)	51,787 (10.9)	65,139 (13.8)
Japan	69,123	53,819 (77.9)	15,304 (22.1)	29,872 (43.2)	23,911 (34.6)	36 (0.1)	6,219 (9.0)	7,419 (10.7)	1,666 (2.4)
Mongolia	4,055	2,727 (67.3)	1,328 (32.7)	1,831 (45.2)	844 (20.8)	52 (1.3)	142 (3.5)	175 (4.3)	1,011 (24.9)
South Korea	16,750	10,107 (60.3)	6,643 (39.7)	6,178 (36.9)	3,928 (23.5)	1 (0.0)	0 (0.0)	0 (0.0)	6,643 (39.7)
Southern Asia	*54,400*	*41,885 (77.0)*	*12,515 (23.0)*	*11,136 (20.5)*	*27,570 (50.7)*	*3,179 (5.8)*	*619 (1.1)*	*777 (1.4)*	*11,119 (20.4)*
Bangladesh	5,582	3,631 (65.0)	1,951 (35.0)	1,851 (33.2)	1,778 (31.9)	2 (0.0)	456 (8.2)	390 (7.0)	1,105 (19.8)
India	21,082	18,024 (85.5)	3,058 (14.5)	2,898 (13.7)	15,124 (71.7)	2 (0.0)	59 (0.3)	41 (0.2)	2,958 (14.0)
Iran	9,807	7,139 (72.8)	2,668 (27.2)	2,756 (28.1)	4,378 (44.6)	5 (0.1)	2 (0.0)	0 (0.0)	2,666 (27.2)
Nepal [a]	7,863	5,590 (71.1)	2,273 (28.9)	2,195 (27.9)	3,394 (43.2)	1 (0.0)	36 (0.5)	273 (3.5)	1,964 (25.0)
Pakistan [b]	2,547	1,929 (75.7)	618 (24.3)	391 (15.4)	1,072 (42.1)	466 (18.3)	66 (2.6)	73 (2.9)	479 (18.8)
Sri Lanka	7,519	5,572 (74.1)	1,947 (25.9)	1,045 (13.9)	1,824 (24.3)	2,703 (35.9)	0 (0.0)	0 (0.0)	1,947 (25.9)
South-East Asia	*44,681*	*28,483 (63.7)*	*16,198 (36.3)*	*15,111 (33.8)*	*12,802 (28.7)*	*570 (1.3)*	*2,052 (4.6)*	*2,839 (6.4)*	*11,307 (25.3)*
Cambodia	4,077	2,539 (62.3)	1,538 (37.7)	1,335 (32.7)	1,158 (28.4)	46 (1.1)	180 (4.4)	80 (2.0)	1,278 (31.3)
Indonesia	7,567	4,384 (57.9)	3,183 (42.1)	2,741 (36.2)	1,622 (21.4)	21 (0.3)	0 (0.0)	0 (0.0)	3,183 (42.1)
Laos [c]	3,216	1,907 (59.3)	1,309 (40.7)	1,345 (41.8)	559 (17.4)	3 (0.1)	430 (13.4)	194 (6.0)	685 (21.3)
Philippines	5,451	3,013 (55.3)	2,438 (44.7)	1,199 (22.0)	1,436 (26.3)	378 (6.9)	371 (6.8)	818 (15.0)	1,249 (22.9)
Singapore	10,317	7,441 (72.1)	2,876 (27.9)	3,867 (37.5)	3,477 (33.7)	97 (0.9)	610 (5.9)	1,010 (9.8)	1,256 (12.2)
Thailand	7,860	5,190 (66.0)	2,670 (34.0)	2,551 (32.5)	2,627 (33.4)	12 (0.2)	461 (5.9)	732 (9.3)	1,477 (18.8)
Vietnam	6,193	4,009 (64.7)	2,184 (35.3)	2,073 (33.5)	1,923 (31.1)	13 (0.2)	0 (0.0)	5 (0.1)	2,179 (35.2)
Oceania-Melanesia-Polynesia	*49,142*	*36,112 (73.5)*	*13,030 (26.5)*	*16,915 (34.4)*	*7,397 (15.1)*	*11800 (24.0)*	*138 (0.3)*	*711 (1.4)*	*12,181 (24.8)*
Australia	33,393	25,500 (76.4)	7,893 (23.6)	12,933 (38.7)	5,144 (15.4)	7,423 (22.2)	17 (0.1)	105 (0.3)	7,771 (23.3)
New Zealand	15,749	10,612 (67.4)	5,137 (32.6)	3,982 (25.3)	2,253 (14.3)	4,377 (27.8)	121 (0.8)	606 (3.8)	4,410 (28.0)
Total	*711,734*	*488,263*	*223,471*	*297,548 (41.8)*	*168,045 (23.6)*	*22,670 (3.2)*	*5,0697 (7.1)*	*63,708 (9.0)*	*109,066 (15.3)*

	(68.6)	*(31.4)*

[a] Data were available for 2012–2017
[b] Data were available for 2010–2016
[c] Data were available for 2011–2017
