# Supplementary material for: Epidemiology and timing of seasonal influenza epidemics in the Asia-Pacific region, 2010–2017: implications for influenza vaccination programs
Source: BMC Public Health. 2019 Mar 21;19:331. doi: 10.1186/s12889-019-6647-y (PMC6429768; doi:10.1186/s12889-019-6647-y)
Supplement: Supplementary file 2 — Table S2 Number and proportion of influenza cases each year overall, by type, and by subtype/lineage. (PDF 16 kb) [file 12889_2019_6647_MOESM2_ESM.pdf]

Supplementary Table 2. Number and proportion of influenza cases each year overall, by type, and by subtype/lineage

		n (% of cases)							
**Year**	**Any influenza**	**Any A**	**Any B**	**A(H3N2)**	**A(H1N1)**	**A (other, not subtyped)**	**B Victoria**	**B Yamagata**	**B not characterized**
2010	73,770	44,850 (60.8)	28,920 (39.2)	16,542 (22.4)	24,108 (32.7)	4,200 (5.7)	2,871 (3.9)	1,759 (2.4)	24,290 (32.9)
2011	48,181	33,401 (69.3)	14,780 (30.7)	10,564 (21.9)	21,232 (44.1)	1,605 (3.3)	2,827 (5.9)	1,047 (2.2)	10,906 (22.6)
2012	73,707	40,929 (55.5)	32,778 (44.5)	34,875 (47.3)	3,916 (5.3)	2,138 (2.9)	10,178 (13.8)	4,523 (6.1)	18,077 (24.5)
2013	55,696	44,148 (79.3)	11,548 (20.7)	25,208 (45.3)	17,756 (31.9)	1,184 (2.1)	937 (1.7)	2,886 (5.2)	7,725 (13.9)
2014	99,293	71,346 (71.9)	27,947 (28.1)	42,340 (42.6)	27,069 (27.3)	1,937 (2.0)	1,395 (1.4)	10,420 (10.5)	16,132 (16.2)
2015	104,312	75,316 (72.2)	28,996 (27.8)	57,426 (55.1)	15,271 (14.6)	2,619 (2.5)	1,482 (1.4)	17,960 (17.2)	9,554 (9.2)
2016	120,381	75,268 (62.5)	45,113 (37.5)	39,459 (32.8)	33,299 (27.7)	2,510 (2.1)	22,910 (19.0)	7,479 (6.2)	14,724 (12.2)
2017	136,394	103,005 (75.5)	33,389 (24.5)	71,134 (52.2)	25,394 (18.6)	6,477 (4.7)	8,097 (5.9)	17,634 (12.9)	7,658 (5.6)
Total	*711,734*	*488,263 (68.6)*	*22,3471 (31.4)*	*297,548 (41.8)*	*168,045 (23.6)*	*22,670 (3.2)*	*50,697 (7.1)*	*63,708 (9.0)*	*109,066 (15.3)*
